# Importance of the neutrophil–lymphocyte ratio and systemic immune‐inflammation index in predicting colorectal pathologies in fecal occult blood‐positive patients

**DOI:** 10.1002/jcla.24878

**Published:** 2023-04-25

**Authors:** Ismail Hasirci, Alpaslan Şahin

**Affiliations:** ^1^ Department of General Surgery Konya City Hospital Konya Turkey

**Keywords:** colonoscopy, colorectal cancer, fecal occult blood, neutrophil/lymphocyte ratio, systemic immune‐inflammation index

## Abstract

**Background:**

The fecal occult blood (FOB) test is one of the primary screening methods for colorectal cancer (CRC). In this study, we aimed to investigate the effect of the neutrophil/lymphocyte ratio (NLR) and systemic immune‐inflammation index (SII) in predicting CRC and other colorectal pathologies in patients with a positive FOB test.

**Methods:**

This retrospective study included patients with a positive FOB test who underwent colonoscopy for the investigation of the etiology. The optimal cutoff levels of NLR and SII for predicting colorectal pathologies were determined using the receiver operating characteristic analysis.

**Results:**

Of the 157 FOB+ patients, 35% were male and 65% were female, with a median age of 59 years. There were 96 patients in Group 1 and 61 patients in Group 2. The mean age of the patients in Group 2 was significantly higher (*p* < 0.001). The rate of male patients was significantly higher in Group 2. NLR and SII were significantly higher in Group 2 than in Group 1 (*p* < 0.001). The area under the curve values of NLR and SII in predicting colorectal pathologies in FOB+ patients were 0.817 and 0.825, respectively. At the cutoff values of 0.689 and 0.795, NLR and SII had a sensitivity of 60.66% and 95.83%, respectively, and a specificity of 95.83% and 100%, respectively.

**Conclusion:**

Neutrophil/lymphocyte ratio and SII can be used as important biomarkers in the early diagnosis of CRC and other colorectal lesions in patients with a positive FOB test.

## Graphical abstract

The fecal occult blood (FOB) test is one of the primary screening methods for CRC. In this study, we aimed to investigate the effect of the neutrophil/lymphocyte ratio (NLR) and systemic immune‐inflammation index (SII) in predicting CRC and other colorectal pathologies in patients with a positive FOB test. This retrospective study included patients with a positive FOB test who underwent colonoscopy for the investigation of the etiology. The optimal cutoff levels of NLR and SII for predicting colorectal pathologies were determined using the receiver operating characteristic analysis. Of the 157 FOB+ patients, 35% were male and 65% were female, with a median age of 59 years. There were 96 patients in Group 1 and 61 patients in Group 2. The mean age of the patients in Group 2 was significantly higher (*p* < 0.001). The rate of male patients was significantly higher in Group 2. NLR and SII were significantly higher in Group 2 than in Group 1 (*p* < 0.001). The area under the curve values of NLR and SII in predicting colorectal pathologies in FOB+ patients were 0.817 and 0.825, respectively. At the cutoff values of 0.689 and 0.795, NLR and SII had a sensitivity of 60.66% and 95.83%, respectively, and a specificity of 95.83% and 100%, respectively. Neutrophil/lymphocyte ratio and SII can be used as important biomarkers in the early diagnosis of CRC and other colorectal lesions in patients with a positive FOB test.

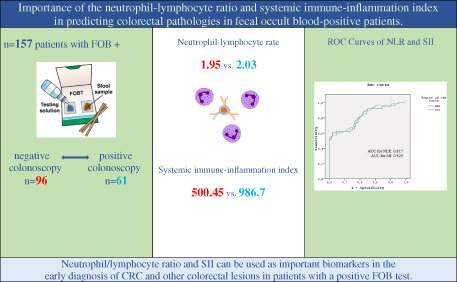

## 1 INTRODUCTION

Colorectal cancer (CRC) is globally the third most common type of cancer and the third largest cause of cancer‐related deaths.^1^, ^2^ The detection of CRC at an early stage allows for the curative endoscopic or surgical treatment of the disease and plays an important role in determining survival. While the 5‐year survival rate remains around 90% with early diagnosis, this rate drops to 13% if the diagnosis is delayed.^3^ The fecal occult blood (FOB) test is used across the world in the early diagnosis of CRC and has been shown to reduce mortality due to CRC by 25%.^4^ In another study, the sensitivity of the FOB test was determined to be 12.9%–79.4% and its specificity ranged from 86.7% to 97.7%.^5^

Systemic inflammation is known to be an important risk factor for cancer development. The indicators of systemic inflammation are mediators such as liver‐derived albumin, circulating leukocytes, and platelets. Neutrophils, monocytes, and platelets increase cancer cell proliferation, invasion, and metastasis.^6^ Lymphocytes, on the other hand, play an important role in tumor defense by inducing cytotoxic cell death and inhibiting tumor cell proliferation and migration.^7^ The presence of systemic inflammation is associated with a poor prognosis as well as being involved in cancer development and progression.^8^

Among the current parameters added to inflammation markers are the neutrophil/lymphocyte ratio (NLR) and the systemic immune‐inflammation index (SII).^9^, ^10^ Studies have shown that as a simple systemic inflammatory marker, the preoperative peripheral blood NLR value is an indicator of prognosis and survival in CRC.^11^ Previous studies have also found SII to be a useful index in determining the prognosis of CRC and hepatocellular carcinoma.^10^, ^12^ However, the possible predictive role of these indices in the diagnosis of colorectal pathologies in FOB‐positive patients has not yet been investigated.

To effectively and sustainably reduce the incidence of CRC and the associated mortality, CRC screening must be optimized. Another primary goal is the development of low‐cost, reliable, and noninvasive screening methods.^13^

In this study, we aimed to investigate the ability of NLR and SII to predict colorectal pathologies by comparing patients with normal findings and those with colorectal pathologies on colonoscopies performed in our clinic due to FOB positivity detected within the scope of the screening program.

## 2 MATERIALS AND METHODS

### 2.1 Trial design

We conducted a retrospective analysis at the surgery department of Konya City Hospital, Turkey. The study protocol was approved by the Ethics Committee of KTO Karatay University Faculty of Medicine (decision number: 2022/019, date: 29.12.2022) and each participant provided their signed informed consent. The “Strengthening the Reporting of Observational Studies in Epidemiology” (STROBE) criteria were used to develop the study protocol.^14^ The study was carried out in conformity with the ethical guidelines of the Declaration of Helsinki.

### 2.2 Participants and eligibility criteria

This study was conducted at the General Surgery Clinic of Konya City Hospital from October 1, 2021 to October 31, 2022. Patients aged over 18 years who presented to our clinic with a positive FOB test performed with the Guaiac method and underwent colonoscopy at the endoscopy unit were retrospectively screened. Patients aged below 18 years, those with inadequate colonoscopic examinations for reasons such as insufficient bowel cleansing, those who only underwent rectosigmoidoscopy, patients with a known disease that could cause gastrointestinal bleeding, known benign anorectal disease, chronic renal and liver failure, or a history of another malignancy, those receiving antiaggregant or anticoagulant treatment or using nonsteroidal anti‐inflammatory drugs, those with incomplete clinical data were excluded. The patients with normal colonoscopy findings were evaluated as having negative colonoscopy results and included in Group 1. Possible bleeding lesions, such as masses and polyps, were accepted as positive colonoscopy results, and these patients were included in Group 2. In Group 2, biopsy samples were obtained from the lesions detected on colonoscopy, and the pathological examination results of these patients were also noted.

### 2.3 Data collection

Patient files, electronic hospital records, outpatient clinic follow‐up records, colonoscopy records, and routine hemogram test results were evaluated. The patients' demographic characteristics and complete blood count parameters were recorded. NLR was determined by dividing the absolute neutrophil count by the absolute lymphocyte count.^15^ PLR was calculated by dividing the absolute platelet count by the absolute lymphocyte count. The mean platelet volume (MPV)/platelet ratio (MPR) was obtained by dividing MPV by the absolute platelet count. SII was calculated by multiplying NLR with the platelet count.^10^

### 2.4 Statistical analysis

In this study, the Statistical Package for the Social Sciences, version 22.0 (SPSS) was used for the statistical analysis of the data. In descriptive statistics, mean, standard deviation, median, minimum, and maximum values were used for continuous variables, and number and percentage values were calculated for discrete variables. The Mann–Whitney *U* test was conducted for the comparison of numerical data between two independent groups, and the chi‐square test was for the comparison of categorical variables. Results were evaluated at the 95% confidence interval, and *p* < 0.05 was defined as statistical significance.

Receiver operating characteristic (ROC) curves were constructed to compare the predictive ability of inflammatory markers and determine their cutoff values. A nomogram was developed according to the contribution of risk factors, and the discrimination and calibration were validated by the ROC and calibration curves. Statistical differences were evaluated at *p* < 0.05.

## 3 RESULTS

A total of 157 FOB+ patients were included in the study (Group 1: 96 patients, Group 2: 61 patients). Thirty‐five percent (*n* = 55) of the patients were male, and 65% were female (*n* = 102). The median age was 59 years. The mean age of the patients in Group 2 was significantly higher (*p* < 0.001). The rate of male patients was significantly higher in Group 2. NLR and SII were significantly higher in Group 2 than in Group 1 (*p* < 0.001). PLR was significantly higher in Group 1 than in Group 2 (*p* < 0.001). The hemoglobin, Platelet level, RDW, CRP, WBC, and MPV values were also similar between the groups (Table 1).

**TABLE 1:** Demographic and laboratory characteristics of the groups.

	Group 1 (*n* = 96)	Group 2 (*n* = 61)	*p* Value
Demographic features			
Age (years)	58 (52–62)	62 (56–69)	**<0.001**
Gender (female/male)	26 (27.1%)	29 (47.5%)	**0.009**
Laboratory tests			
NLR [median (min–max)]	1.95 (1.3–2.5)	2.03 (1.4–2.9)	**<0.001**
PLR [median (min–max)]	124.6 (93.73–156.9)	116.4 (96.7–164.4)	**<0.001**
Hemoglobin (gr/dL)	13.6 (12.4–14.5)	14.5 (13.1–15.1)	**0.012**
Platelet level (10^9^/L)	272 (227–321)	267 (109–510)	0.339
RDW (%)	13.3 (12.8–14.7)	13.3 (12.85–13.8)	0.537
CRP (mg/L)	4.83 (3.05–12)	3.23 (3.03–12.95)	0.460
WBC (10^9^/L)	7.20 (6.21–7.20)	7.4 (6.50–9.05)	0.470
MPV (fL)	10.65 (9.9–11.1)	10.41 (9.82–11.05)	0.541
SII [median (min‐max)]	500.45 (171.29–1126.48)	986.7 (249.4–3755)	**<0.001**

*Note:* Data are presented as median (Q1‐Q3) or number (percentage). The significance of bold values is *p* < 0.05.

Abbreviations: CRP, C‐reactive protein; MPV, mean platelet volume; NLR, neutrophil/lymphocyte ratio; PLR, platelet/lymphocyte ratio; RDW, red cell distribution width; SII, systemic immune‐inflammation index; WBC, white blood cell.

Table 2 shows the colorectal pathologies detected on colonoscopies. The colonoscopic examination revealed colorectal pathologies in 61 (37.6%) of the patients in Group 2. When the biopsy results of these patients were examined, 18 (29.5%) of the 61 patients had hyperplastic polyps, 25 (40.9%) had tubular adenomas, two (3.2%) had adenocarcinomas, and nine (14.7%) had colitis.

**TABLE 2:** Distribution of colorectal pathologies.

Pathology	*n*/%
Hyperplastic polyp	18 (29.5%)
Adenomatous polyp.	
Tubular adenoma	25 (40.9%)
Tubulovillous adenoma	4 (6.5%)
Villous adenoma	3 (4.9%)
Adenocarcinoma	2 (3.2%)
Others (colitis, etc.)	9 (14.7%)

**FIGURE 1:**
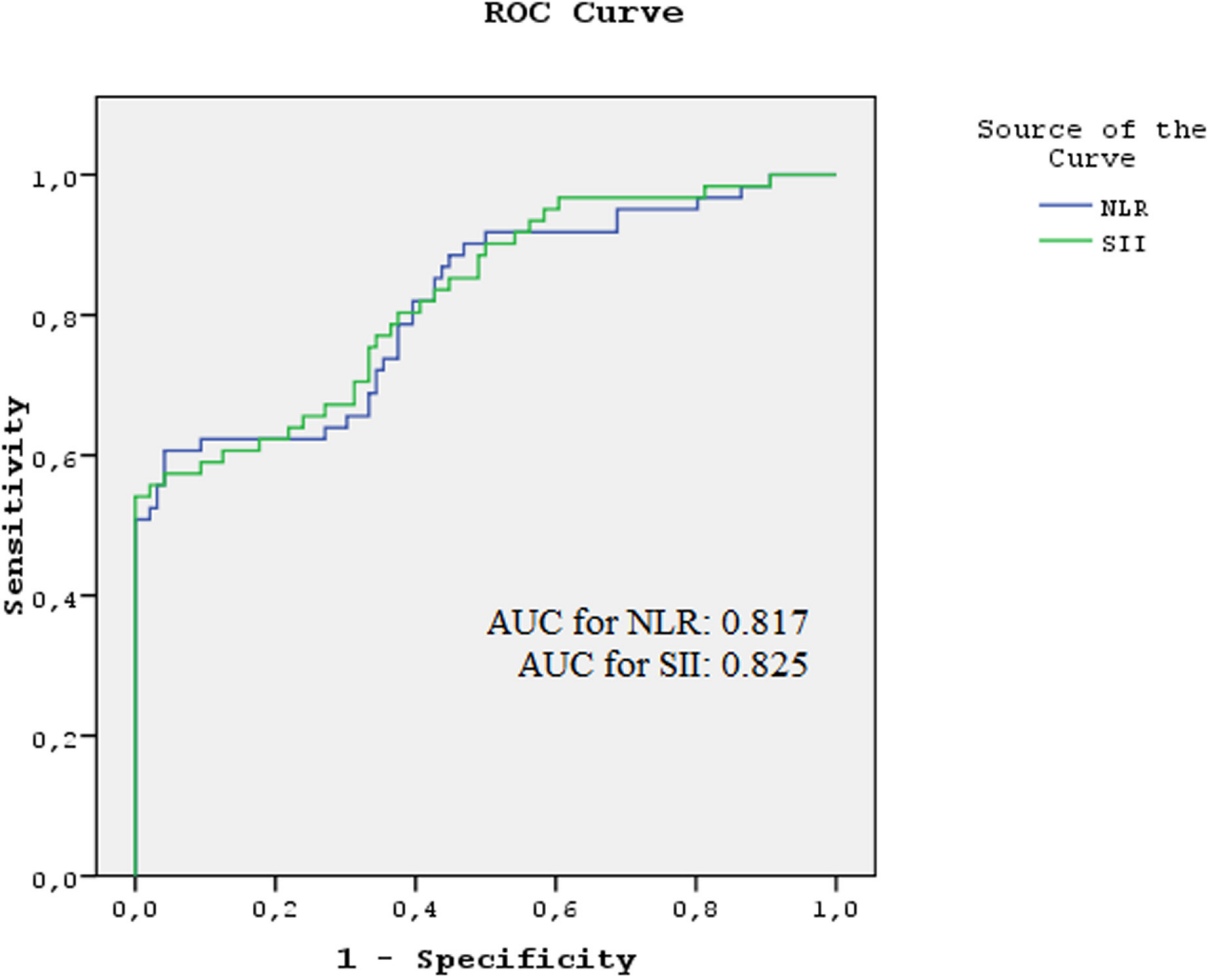
ROC curves of NLR and SII in the detection of colorectal pathologies. NLR, neutrophil/lymphocyte ratio; ROC, receiver operating characteristic; SII, systemic immune‐inflammation index.

Figure 1 and Table 3 present the results of the ROC analysis performed to determine the power of NLR and SII in detecting colorectal pathologies in FOB+ patients. The AUC value of NLR and SII for predicting colorectal pathologies was determined to be 0.817 and 0.825. The AUC values of NLR and SII in the prediction of colorectal pathologies were 0.817 and 0.825, respectively. When the cutoff values of NLR, SII, and PLR were taken as 3.03, 639.96, and 163.51, respectively, these parameters were found to have sensitivity values of 60.66%, 54.1%, and 26.23%, respectively; specificity values of 95.83%, 100%, and 82.29%, respectively; positive predictive values of 90.2%, 100%, and 48.5%, respectively; and negative predictive values of 79.3%, 77.4%, and 63.7%, respectively.

**TABLE 3:** Overall accuracies of laboratory parameters in the differentiation of colorectal pathologies (%).

	Sensitivity	Specificity	PPV	NPV	Cutoff	Youden's index
WBC	63.93	47.92	43.80	67.60	7.02	11.85
CRP	46.15	75.00	54.50	68.20	3.10	21.15
MPV	95.00	13.54	40.70	81.30	11.50	8.54
NLR	60.66	95.83	90.20	79.30	3.03	56.49
PLR	26.23	82.29	48.50	63.70	163.51	8.52
SII	54.10	100	100	77.4	639.96	54.10

Abbreviations: CRP, C‐reactive protein; MPV, mean platelet volume; NLR, neutrophil/lymphocyte ratio; NPV, negative predictive value; PLR, platelet/lymphocyte ratio; PPD, positive predictive value; RDW, red cell distribution width; SII, systemic immune‐inflammation index; WBC, white blood cell.

## 4 DISCUSSION

The detection of CRC at an early stage allows for the curative endoscopic or surgical treatment of the disease and constitutes the most important factor in determining survival. In this study, we investigated the role of NLR and SII and other hemogram parameters in detecting colorectal pathologies in FOB+ patients. NLR and SII were found to be significant parameters for predicting colorectal pathologies in these patients.

In recent years, the prevalence of CRC has increased at an alarming rate on a global scale. CRC is the third largest cause of cancer‐related deaths across the world for both sexes. Due to the ongoing increase in the rate of CRC, it is estimated that by 2040, the number of new colon cancer cases will have reached 1.92 million, new rectal cancer cases 1.16 million, and new anal cancer cases 78,000.^2^ The most important risk factor for CRC is usually age, and the mean age at diagnosis is 68 years.^16^ In the current study, the median age of the patients was 59 years.

Colorectal cancer screening increases the early detection of CRC and allows for the complete resection of precancerous lesions. Thus, it is considered to be the most effective way to halt the progression of CRC. In clinical practice, a two‐stage screening technique is frequently used,^17^ in which the first stage refers to the use of very sensitive stool‐based tests to identify blood and molecular markers in the stool and the second involves visual examinations to visualize the colon and rectum.^18^

Stool‐based tests, such as the FOB test based on the Guaiac method, fecal immunochemical tests, and stool DNA tests are included in community screening programs as noninvasive screening methods that do not require any special preparation. If the test result is positive, invasive endoscopic procedures, such as colonoscopy and sigmoidoscopy, are undertaken to confirm the pathological results.^19^, ^20^

Colorectal cancer screening should be optimized to effectively and sustainably reduce the incidence of CRC and the associated mortality rate.^21^ In daily clinical practice, carcinoembryonic antigen and carbohydrate antigen are widely used biomarkers for the detection and monitoring of CRC, but there is still a need for more reliable biomarkers due to the insufficient sensitivity and low organ specificity of these parameters.^22^

New tests are also being developed for CRC screening.^13^ The United States Food and Drug Administration has approved a blood‐based test that measures Septin 9 methylation for CRC screening in average‐risk individuals aged 50 years and older who have rejected previous CRC screening techniques. However, due to the low sensitivity of this method and the lack of longitudinal and comparative data on its performance, this test is not currently the best screening method.^21^ In this context, the parameters investigated in our study are more favorable since they were obtained from the complete blood count analysis, which is an inexpensive, widely preferred, and easily accessible method.

The American Cancer Society recommends that people aged over 45 years undergo routine CRC screening.^21^ In Turkey, in the CRC screening of men and women over the age of 50, the FOB test is recommended every 2 years and a colonoscopic examination every 10 years.^23^ In the current study, the median age of the patients was 59 years, and all patients underwent colonoscopies due to FOB positivity.

Certain benefits can be obtained when stool‐based tests are compared with radiographic or endoscopic methods. Stool‐based tests are easy to perform at home and do not significantly interfere with daily activities or put the patient at risk. The FOB test can also be performed by a nonphysician.^24^ However, since most polyps do not bleed, they cannot be detected by stool‐based tests.^25^ In the literature, it has been reported that colonoscopic findings are normal in approximately 60% of patients with a positive FOB test.^26^ Consistently, in our study, no colorectal pathology was detected in the colonoscopies of 63% of the patients.

In a study conducted by Bjerrum et al.^26^ in a Danish population, CRC was detected at a rate of approximately 5% and adenomas at 15% in the colonoscopic screening of 26,123 FOB+ individuals. In our study, CRC was present in 1.2% and adenomas in 30.6% of the 157 patients with a positive FOB test. This result shows that the FOB+ patients win our sample consisted of those with a high rate of adenomas, unlike the literature.

Neutrophil–lymphocyte ratio is calculated by dividing the absolute neutrophil count by the lymphocyte count. This ratio is accepted as a parameter that shows the negative effects of both high neutrophils, reflecting acute inflammation, and low lymphocyte levels, representing physiological stress.^25^ In the early diagnosis of CRC, the addition of current biomarkers such as NLR and SII, which show inflammation and immune status, to inflammation markers has been on the agenda in recent years.^27^, ^28^

In many studies, NLR has been shown to be an independent diagnostic and prognostic factor in CRC.^29^, ^30^ In studies investigating the utility of NLR in the prediction of the prognosis of patients with CRC, cutoff values have been determined to be far above the diagnostic values. For example, in a study by Dimitriou et al.,^31^ evaluating 296 patients with stage 2 CRC, the cutoff value of NLR for the prediction of overall survival was reported to be 4.7. In other studies in the literature, the cutoff value of NLR was determined to be 5 among 440 CRC cases presenting with liver metastases^32^ and 3 in 3857 patients with CRC.^33^ Lalosevic et al.^29^ found the cutoff value of NLR to be significantly higher in patients with CRC (2.15), compared with healthy individuals In another study, Ainsa et al.^28^ observed that NLR had an optimal cutoff value of 2.28 (sensitivity: 59.81%, specificity: 81.31%) for potential diagnostic use in CRC. In the current study, the optimum cutoff value of NLR was 3.03 (sensitivity: 60.66%, specificity: 95.83%) for predicting colorectal pathologies in FOB+ patients. The NLR cutoff value we obtained in our study is similar to the results reported in the literature.

Systemic immune inflammation index is obtained by multiplying NLR with the platelet count. SII was first shown to be a strong prognostic marker in hepatocellular carcinoma in a 2014 publication.^10^ Since then, the diagnostic and prognostic value of SII has also been demonstrated in CRC.^10^, ^28^ In addition, SII has been reported to be an indicator of metastatic CRC in patients receiving first‐line chemotherapy with bevacizumab.^34^ In a study by Ainsa et al.,^28^ the optimal cutoff value of SII was found to be 616.50 (sensitivity: 50.47%, specificity: 90.65%) for its potential diagnostic use in CRC. Similarly, in our study, the optimum cutoff value of SII was observed to be 639.96 (sensitivity: 54.1%, specificity: 100%) in the prediction of colorectal pathologies in FOB+ patients.

This is the first study in the current literature to evaluate the use of the hemogram parameters NLR and SII in the prediction of CRC in patients with a positive FOB test. The results of the study revealed that these parameters had statistically significant predictive values. However, our study was limited by its retrospective and single‐center design.

## 5 CONCLUSION

The early diagnosis of CRC is of paramount importance. As simple, inexpensive, and easily accessible laboratory parameters, NLR and SII, are suitable for routine clinical use as important biomarkers in the prediction of colorectal pathologies in the presence of a positive FOB test performed for screening purposes. The use of these two biomarkers will provide benefits in the early diagnosis and treatment of patients with CRC.

## CONFLICT OF INTEREST STATEMENT

The authors declare that they have no conflict of interest.

## Data Availability

This published paper includes all data generated or analysed during this study. If more information is desired, it can be obtained by contacting the corresponding author.
